# An isomorphic mapping hypothesis of the grid representation

**DOI:** 10.1098/rstb.2012.0521

**Published:** 2014-02-05

**Authors:** Michael Brecht, Saikat Ray, Andrea Burgalossi, Qiusong Tang, Helene Schmidt, Robert Naumann

**Affiliations:** 1 Bernstein Center for Computational Neuroscience, Humboldt University of Berlin, Philippstrasse 13 Haus 6, 10115 Berlin, Germany; 2 Werner Reichardt Centre for Integrative Neuroscience, Otfried-Müller-Strasse 25, Tübingen 72076, Germany

**Keywords:** grid cell, spatial representation, border cell, interference model, attractor model

## Abstract

We introduce a grid cell microcircuit hypothesis. We propose the ‘grid in the world’ (evident in grid cell discharges) is generated by a ‘grid in the cortex’. This cortical grid is formed by patches of calbindin-positive pyramidal neurons in layer 2 of medial entorhinal cortex (MEC). Our isomorphic mapping hypothesis assumes three types of isomorphism: (i) metric correspondence of neural space (the two-dimensional cortical sheet) and the external two-dimensional space within patches; (ii) isomorphism between cellular connectivity matrix and firing field; (iii) isomorphism between single cell and population activity. Each patch is a grid cell lattice arranged in a two-dimensional map of space with a neural : external scale of approximately 1 : 2000 in the dorsal part of rat MEC. The lattice behaves like an excitable medium with neighbouring grid cells exciting each other. Spatial scale is implemented as an intrinsic scaling factor for neural propagation speed. This factor varies along the dorsoventral cortical axis. A connectivity scheme of the grid system is described. Head direction input specifies the direction of activity propagation. We extend the theory to neurons between grid patches and predict a rare discharge pattern (inverted grid cells) and the relative location and proportion of grid cells and spatial band cells.

## 1. Introduction

The observation of grid cell activity by the Mosers and co-workers [1] is one of the most striking findings about the neural representation of space. Grid cells have been described mainly but not exclusively [2] in the medial entorhinal cortex (MEC) and form a robust allocentric representation of space [1]. As indicated by their name, grid cells discharge in a firing field that is organized in a hexagonal (or triangular) grid. The orientation of the grid is similar across cells locally [1] and perhaps also globally [3–5]. The phase of grid cells varies locally [1]. Grid spacing—the distance from firing node to firing node—varies systematically across the entorhinal cortex and is small dorsally (approx. 30 cm) and large ventrally (approx. 3 m) [6]. A consensus has emerged that, of the different layers of entorhinal cortex, layer 2 contains the largest fraction of and the clearest grid cells [2]. Besides grid cells, investigators have described the so-called ‘border’ [7] or ‘boundary’ [8] or ‘boundary vector cells’ [9,10]; these cells do not fire in restricted discharge fields, but instead discharge along borders [7,8] in a certain direction in the room (wherever this border is), and finally, spatial band cells have been described, which discharge in a band pattern across the room [11].

Immediately following the discovery of grid cells, a large number of grid cell models have been proposed (for review, see [12]). These can be divided into oscillatory interference models [13–17] and attractor models [18–20]. To start with, we outline in what ways the approach taken here differs from previous modelling work. The key novelty of the theory presented here is that it focuses on the modular architecture of the MEC. The inspiration for the hypothesis does not come from temporal dynamics (i.e. phase precession) or attractor network theory [21], but it comes from the modular anatomy of layer 2 of the entorhinal cortex [22–24]. We focus on the question of how to connect grid cells across spatial scales, so that they form a coherent look-up table of where the animal moves in space. The model is derived from three isomorphism assumptions, of which only one (the correspondence of neural and external two-dimensional space) is very strong and essential to the model. It is possible to explain grid cell and spatial band cell activity in terms of a wiring diagram that specifies connectivity in layer 2 at a level of approximately 5 µm.

## 2. The isomorphic mapping hypothesis

### (a) Overview

We first provide a minimal sketch of the operation of the grid cell system in layer 2 of the rat MEC. The isomorphic mapping hypothesis is inspired by the similarity between the pattern of patches in the MEC and grid cell discharge patterns in space [24]. It is suggested that layer 2 of the MEC consists of repeating modules, namely grid cell patches of calbindin-positive pyramidal neurons [25]. According to the hypothesis, we assume three types of isomorphism: (i) a metric correspondence of neural space and the external space within patches; (ii) an isomorphism between connectivity matrix of a grid cell and its firing field; (iii) an isomorphism between single cell and population activity within a patch. Following the introduction of the hypothesis, we will specify a connectivity scheme for layer 2 of the MEC and discuss the propagation of activity during spatial navigation. We conclude by an extension of the theory to non-metric discharge patterns, such as spatial band cells.

### (b) Layer 2 anatomy and grid cell discharge

**Figure 1.**
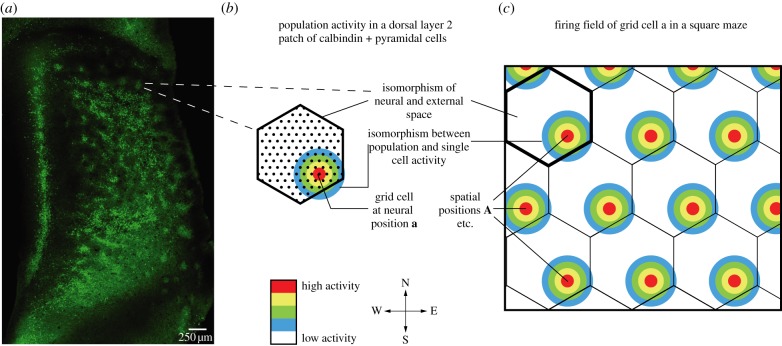
Experimental data, isomorphic mapping of space, single cell and population activity. (*a*) Tangential section of MEC processed for calbindin immunoreactivity showing grid-like arrangement of calbindin-positive patches in layer 2. Detailed description and quantification of morphological arrangement by grid scores is provided in [24]. (*b*) Colour-coded population activity of calbindin-positive pyramidal cells (black dots) in a layer 2 patch. (*c*) Firing field of a grid cell a in a square maze.

A key insight has been the finding that principal neurons in rat MEC can be divided into two classes: calbindin-positive pyramidal-like neurons that project extra-hippocampally, and reelin-positive stellate cells that project to the dentate gyrus [26]. Layer 2 of the MEC of many species contains patches [22,23]. It appears that layer 2 of rat MEC contains multiple systems of patches, one revealed by staining for cytochrome-oxidase activity [22] and one revealed by staining for the calcium-binding protein calbindin; their interrelation is currently not known. Here, we focus on the patches formed by calbindin-positive pyramidal cells [25], which are arranged in a regular pattern similar to grid cell discharge patterns [24]. Figure 1*a* shows the grid-like arrangement of these patches in layer 2 of the MEC. Calbindin patches have a diameter of about 150 µm and are arranged in a regular array (figure 1*a*, see [24] for details). Different from grid cell activity and from patches revealed by cytochrome-oxidase activity staining [22], there is little or no change in calbindin-patch spacing from dorsal to ventral cortex [23]. Here, we will pursue the hypothesis that the similarity of the calbindin-patch arrangement and grid cell discharge reflects a deep isomorphism between the neural and external mapping of space.

### (c) Concept of the isomorphic mapping hypothesis

According to our hypothesis, we assume three types of isomorphism: (i) a metric correspondence of neural space (the two-dimensional cortical plane) and the external two-dimensional Cartesian space within calbindin grid cell patches; (ii) an isomorphism between the connectivity matrix of a grid cell and its firing field (i.e. firing rate at a certain site corresponds to strength of connections to cells, whose firing rate peaks at this site); (iii) an isomorphism between single grid cell and population grid activity within a patch. All these assumptions are simplifications, and only assumption (i) is very strong and essential to the theory. Assumptions (ii) and (iii) greatly simplify the interpretation of entorhinal circuits, but they are less critical to the theory and can easily be relaxed.

Figure 1 describes how these assumptions actually work for a dorsal grid cell patch (figure 1*b*). A layer 2 grid cell patch, seen in a top view, is a metric representation of repeating segments of real space (figure 1*c*). We show a patch in the shape of a hexagon (figure 1*b*), which makes it easy to illustrate how real space (figure 1*c*) is represented and tiled up by the grid patch. The hexagonal shape of a grid patch is not essential to our theory, however, and anatomically calbindin-patches look typically circular. While the hexagonal shape of a grid patch is not essential to the model, the arrangement of multiple grid patches in a hexagonal grid is of significance for the connectivity scheme proposed later and our ideas how spatial band cells might be generated from grid cell inputs (see below). Spatial direction applies in both the real and neural space. This is our first isomorphism assumption. Figure 1*b* illustrates, in a colour-coded population, firing rate in the patch at a set of positions **a** in real space, whereas figure 1*c* illustrates, in a colour-coded single neuron, firing rate of a grid cell, which has its peak firing rate at position **A**. The correspondence of single grid cell and population grid activity illustrated here is our third isomorphism assumption.

**Figure 2.**
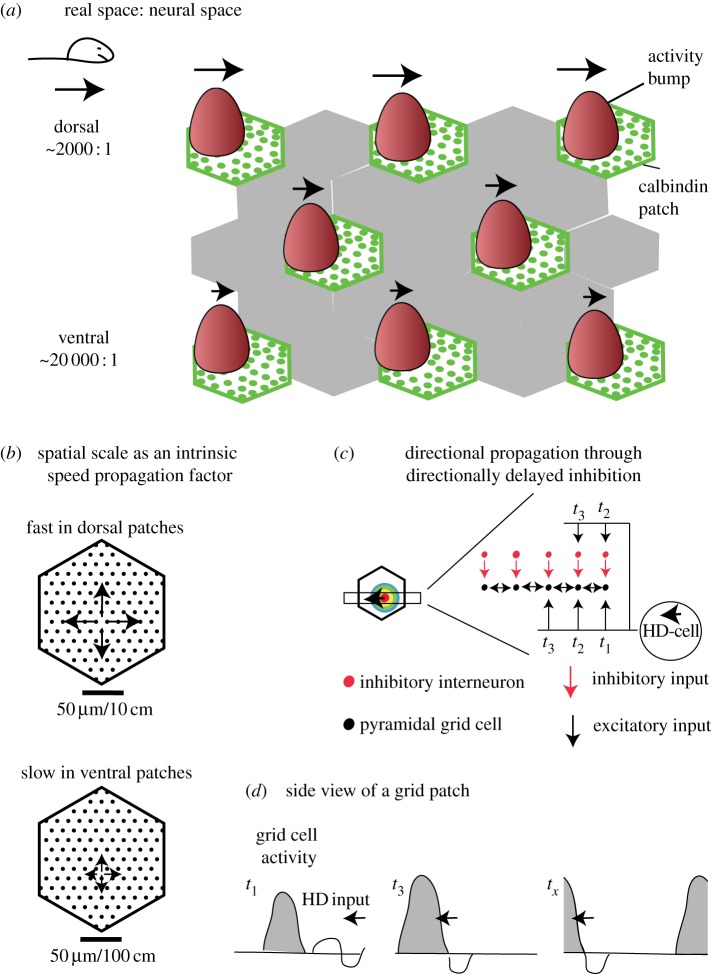
Spatial scale and propagation of activity through patches. (*a*) Overview of the operation of multiple grid patch in spatial navigation. As the animal runs from left to right (schematic on the left), bumps of activity move through medial entorhinal grid patches from left to right (right part). Although the position and speed of these bumps of activity are the same in laterally neighbouring patches, this is not the case for patches at different dorsoventral positions. (*b*) Interpretation of spatial scale as an intrinsic speed propagation factor. (*c*) Head direction input is thought to specify the direction of propagation of population activity. One (of many possible) implementation scheme of propagation of activity by directionally delayed inhibition; the model does not specify how the velocity signal is implemented in the medial entorhinal cortex. (*d*) Side view of the propagation of activity through a grid patch at the time points specified in (*c*). Head direction input (the squiggle) sweeps across the entire patch, but inhibition (the downward-going part of the squiggle) is delayed in the heading direction relative to excitation (the upward-going part of the squiggle). Hence the hill moves forward.

Figure 1 described grid activity in a single calbindin patch. An overview of the operation of multiple grid patches is provided in figure 2*a*. It can be seen that different from conventional attractor models there is not one but many ‘bumps of activity’ [18–20]. Specifically, there is one bump of activity in each grid patch. As the animal moves from left to right (figure 2*a*, left), these bumps of activity move from left to right in the entorhinal cortex (figure 2*a*, right). Although these bumps of activity are at analogous positions and move at identical speed in laterally neighbouring patches, the bumps of activity move at different speeds in patches at different dorsoventral positions.

### (d) The interpretation of spatial scale as an intrinsic speed propagation factor

Figure 1 illustrates neural activity in a grid patch in dorsal MEC (figure 1*b*) and in real space (figure 1*c*). A calbindin patch has a diameter of 150 µm [24] and in dorsal MEC firing field spacings of around 30 cm have been reported [1]. Our isomorphism assumptions thus allow us to deduce a 1 : 2000 scaling of neural to external spacing in dorsal MEC. It is known, however, that grid spacing in ventral entorhinal cortex is about 10 times larger, whereas anatomical analysis shows that patch spacing of calbindin patches is similar or the same in dorsal and ventral cortex. If we want to hold up our isomorphism assumption, then we need to assume that ventral patches intrinsically differ in spatial scale (figure 2*a,b*). Specifically, we assume that there is an intrinsic difference in propagation speed of neural activity between dorsal and ventral patches (figure 2*b*). Note that the actual speed, with which activity travels through the patch, depends also on the animal's velocity in space. Spatial scale is therefore not interpreted as an absolute propagation speed of neural activity but rather as an intrinsic speed propagation factor to be multiplied with the animal's velocity. The idea that neural propagation of activity differs between dorsal and ventral entorhinal cortex is in line with experimental evidence on dorsal/ventral differences in intrinsic properties [16], synaptic transmission and neuronal integration [27–29].

These assumptions predict fast neural propagation in dorsal patches (a spatial scale of approx. 1 : 2000) and slow neural propagation in ventral patches (a spatial scale of approx. 1 : 20 000). What do these numbers mean in neural terms? At a standard running speed of 10 cm s^−1^, a rat will cross a dorsal patch in 3 s and will cover 1.14 cm in one theta cycle. In external space, 1.14 cm corresponds to 5.7 µm in neural space. If one imagines the about 120 calbindin-positive pyramidal cells in one plane (the patch has an area of about 15 000 µm^2^), then they have a spacing of approximately 11 µm [24]. Thus, we predict that at an average running speed population activity in a dorsal patch advances approximately 0.5 cells per theta cycle. The propagation of neural activity will be treated in more detail below.

### (e) Propagation of activity during spatial navigation

The propagation of activity through a grid patch is what leads to grid cell firing, but several aspects of this process are not yet understood. Figure 2*c* illustrates how head direction information drives the propagation of activity through the patch. We assume that the grid cell activity rests statically at a low level that is exactly self-sustaining in the absence of head direction input. While it seems fairly clear that head direction inputs control the direction of propagation activity, how this exactly occurs is not predicted by the theory. To give the reader a better idea how this may occur, we specify one possible implementation of head direction input to grid patches in figure 2*c,d*, but we emphasize that this scheme is not essential to our theory. As shown in figure 2*c*, directionally delayed inhibition activated by head direction inputs can be used to push the population activity forward (figure 2*d*). The local and long-range wrap-around connectivity between grid cell patches (see §2*f*) assures that the grid activity peak wraps around in time (figure 2*d,* right). The velocity signal could involve either the head direction cells or could be implemented through a higher grid cells activity with increasing velocity, which will also lead to faster propagation. Most likely, velocity is implemented in multiple ways in the grid/head direction circuit.

### (f) Connectivity scheme

**Figure 3.**
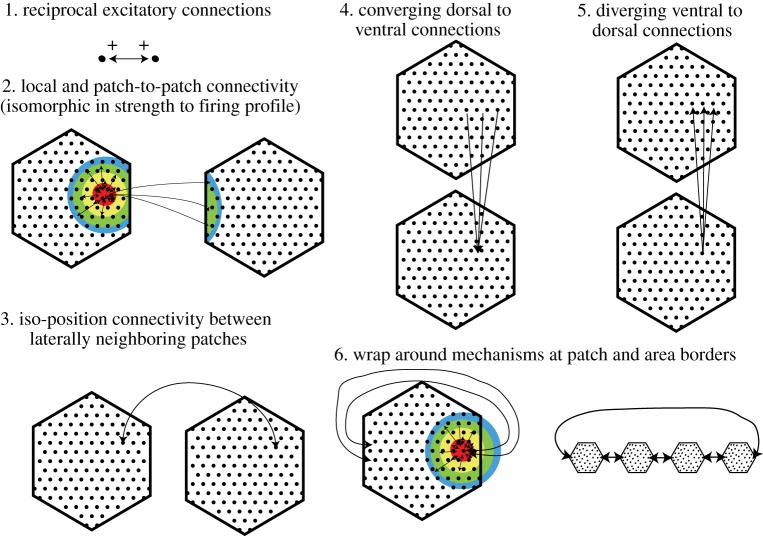
Connectivity rules for the grid system. Connectivity rules proposed for the patches (black hexagons). Connections are illustrated by arrows and are always shown for one cell (black dot) only, but all cells are thought to make the same connections.

The core of our hypothesis is to devise a connectivity scheme that generates grid cell discharges and relates spatial discharge in an unambiguous manner across spatial scales and grid cell patches. We assume that all patches have the same grid orientation. Patches at the same dorsoventral height of MEC are thought to have the same grid map. A set of six connectivity rules that connect grid cells within and across patches is illustrated in figure 3: (i) we assume only excitatory, reciprocal and symmetric connections that are the same between all grid cells. (ii) Within the patch, we assume a correspondence between the connectivity matrix of a grid cell and its firing field; this is our second isomorphism assumption. Second, we assume long-range connectivity that extends symmetrically into neighbouring patches. (iii) We assume isoposition connectivity between patches of the same spatial scale within and across hemispheres. This ensures a collective operation and mutual reinforcement of positional information between neighbouring grid patches. (iv) We assume converging connections from dorsal to ventral patches, because of the change from larger to smaller scale from dorsal to ventral. (v) We assume diverging connections from ventral to dorsal patches, because of the change from smaller to larger spatial scale from ventral to dorsal. (vi) One also needs to invoke wrap-around mechanisms at patch borders, otherwise the activity will die out when the wave of activity hits such a border. We illustrate two types of wrap-around, namely within patches or long-range between patches. This set of rules is rather simple but specifies connectivity in the entire grid cell system. This connectivity ensures a collective and coherent operation of grid cells across spatial scales and across the MEC.

Is this connectivity scheme compatible with the experimental evidence? The mutual excitatory connectivity between layer 2 pyramidal cells postulated here differs sharply from the connectivity observed in paired recording studies of layer 2 stellate neurons, which show almost no mutual excitatory connections and which are coupled by strong disynaptic inhibition [28,30]. Morphological studies [31] have demonstrated that both stellate and pyramidal cells have axon collaterals terminating in layer 2. Uncaging studies [32,33] have revealed evidence for massive excitatory inputs from layer 2 onto both layer 2 pyramidal and stellate neurons, which may originate from layer 2 pyramidal cells since stellate neurons do not seem to form such connections, at least according to the evidence cited above [28,30].

The fact that even same-sized grids can have deviations, such as slightly different tilts in their main axes [5], poses challenges for the connectivity scheme proposed here. In such cases, the proposed isoposition connectivity will not be optimal. We suggest that in such cases a slightly modified connectivity (such as mapping the horizontal axis of one patch to an oblique axis of another patch) will be better than isoposition connectivity. Because such deviations in axes of same-sized grids seem to be stable over months, we argue that such deviations could be learnt.

Grids of different scales shift relative to each other in situations that cause place cell remapping [34]. We think that the modular (patchy) arrangement of grid circuits might underlie the ability of these circuits to functionally dissociate and move relative to each other. Such dissociations pose challenges for the long-range connectivity between patches. This problem might preclude having long-range connectivity between such functionally dissociating patches. Alternatively, this problem requires flexibility in the respective long-range connectivity. The problem could be solved by dynamically switching off long-range connections or by the environment-specific relearning of long-range connectivity.

### (g) Extension of theory to neurons between grid patches

**Figure 4.**
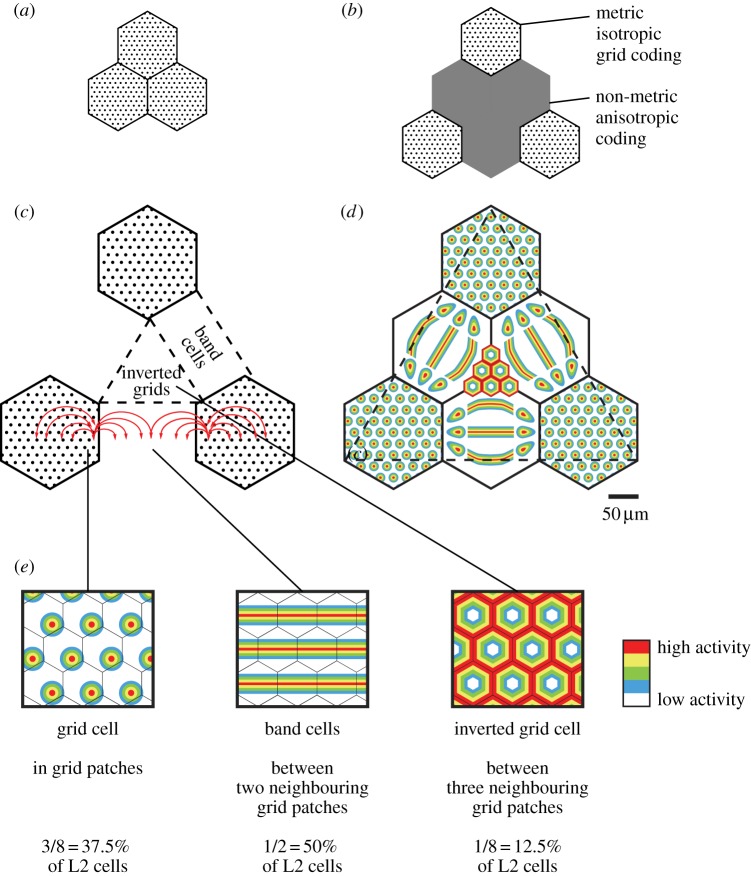
Extension of the theory beyond grid patches. (*a*) Directly adjacent grid patches (black hexagons) would allow the simplest connectivity scheme for the grid system. (*b*) In reality, grid patches are displaced from each other opening up an anisotropic non-metric coding space. (*c*) The theory is extended beyond grid patches (black hexagons) by assuming unilateral but otherwise identical connections from grid cells (black dots) into the non-grid space as into grid patches. The assumed connections are illustrated for two cells in neighbouring grid patches along one dimension. In reality, they are thought to be formed by all grid cells and extend radially in two-dimensional space. (*d,e*) Prediction of spatial firing patterns across layer 2 in a continuous scheme (*d*) or as specific cell types (*e*). Grid cell discharges shown in the square maze (*e*, left) occur in grid patches. Spatial band cell discharges shown in the square maze (*e*, middle) occur between two neighbouring grid patches, because spatial input along the axis connecting the two patches stays the same, but falls off orthogonally to this axis with distance. Inverted grid cells are a novel discharge pattern predicted by the theory and shown in the square maze (*e*, right). Inverted cell discharges are firing fields with ‘firing holes’ arranged in a hexagonal grid with firing peaking between ‘hole’ vertices. Inverted grids occur between three neighbouring grid patches, because cells in this region are close to grid patch borders, but far from grid patch centres. The proportions of cell types given in *e* were predicted by graphical triangulation using the dashed equilateral triangle superimposed on *d*.

This brief account of the isomorphic mapping hypothesis explains many features of grid cell activity, such as iterative firing and spatially coherent activity across scales. The most simple connectivity scheme to implement the entire theory outlined so far would be to arrange grid patches directly adjacent to each other, as depicted in figure 4*a*. As we have seen in the description of the patchy anatomy, this is not the case, however. Instead, putative grid patches are displaced from each other as shown in figure 4*b*.

We now extend our hypothesis to include this space between the grid patches in our theory. With two assumptions, one can also predict spatial firing patterns in this region. These assumptions are (i) cells in this region receive the same grid cell input as grid cell neighbours, but they do not return connections, and (ii) there is no recurrent connectivity in this region. These assumptions imply that the area outside of the patches is downstream from the grid system and that there is no metric spatial firing in between grid patches, because it is the symmetric connectivity of cells in grid patches which guarantees such a code (figure 4*b*).

What then, are the firing fields of cells between the grid patches? We approach this issue in figure 4*c*, where we show the connections of two grid cells in neighbouring patches in one dimension. The entire connectivity matrix would involve all grid cells, which make radial connections in two dimensions. Figure 4*d,e* shows schematically the resulting spatial discharge profiles at various regions in layer 2 of the MEC. Figure 4*d* also shows that the model predicts a gradual shift from grid-like to band-like firing patterns as one moves out from grid patches. In cells in the transition zone from grid-like to band-like firing, varying inputs from neighbouring grid and band cells might cause a flipping back and forth between such discharge patterns as observed recently [11].

#### (i) Grid cells

As discussed before and shown in figure 4*e*, grid cells are observed in grid patches.

#### (ii) Spatial band cells

We predict that exactly between two neighbouring patches, one will observe cells that discharge in spatial bands. This band-like firing pattern comes about because the input from the two neighbouring patches will be the same at this point (figure 4*c*), and the neural activity will be maximal and the same along a line connecting this position and the two neighbouring patches. Along the room axis orthogonal to this connecting line, activity falls off, because the distance of grid cells to this position increases along this axis.

One can also understand how spatial band cells come about in our model, if one considers the dynamical scenario outlined in figure 2*a*. Because cells in the grey area between grid patches receive their inputs from grid patches and input strength from each grid cell falls off with distance, their activity will increase the closer they are to the peak of the activity bumps in grid patches. As the animal runs from left to right (figure 2*a*), the cells between two grid patches in the top row of figure 2*a* will see a constant input level. This constant input level comes about because inputs from the left-hand neighbouring grid patch increase as the activity bump approaches, but at the same time inputs from the right-hand neighbouring grid patch decrease as the activity bump moves away. If the animal runs from top to bottom, however, then input levels of the cells between two grid patches in the top row of figure 2*a* will vary. The cells will see maximum input when the peak of the activity bump in the neighbouring grid patches is exactly lateral from them, and a drop of inputs when the animal moves further up or down. Thus, these cells between two grid patches in the top row of figure 2*a* will fire in a spatial band extending from left to right.

#### (iii) Inverted grid cells

Based on the connectivity considerations shown in figure 4*c*, a novel discharge pattern—inverted grid cells (see also [28])—can be predicted, which should be found in the space between three neighbouring grid patches. This discharge pattern emerges because cells in this region are close to grid cells at patch borders, but distant to patch centres. If one assumes an equal cell density and graphically divides up the areas representing grid cells, band cells and inverted grid cells, one can predict the relative frequencies of these discharge patterns (figure 4*e*, bottom). Again, one can also understand how inverted cells come about in our model if one considers the dynamical scenario outlined in figure 2*a*.

Finally, we would like to add that the spatial firing profiles observed between grid patches depend fairly strongly on the connection parameters, such as radial spread of connections. This parameter dependence differs from the grid connectivity. Parameter dependence arises because we are dealing here with unilateral, asymmetric connections. For example, inverted grid cells come about only if not too long a radial connectivity is chosen; otherwise, one observes very little spatial modulation in this region of the entorhinal cortex. This is an important consideration because there is evidence for a large number of spatially non-modulated cells in the MEC [35], whereas there is no experimental evidence so far for inverted grid cells.

Are the fractions of grid and band cells predicted by this model compatible with published results? There are discrepancies between the reported percentages of grid cells in superficial layer MEC in the literature. Older studies report large fractions of grid cells [36,2], which would be incompatible with our theory. Most recent publications report an estimate of grid cells in L2 of around one-third [11,37,35] or even lower [37], numbers possibly compatible with our hypothesis. Further, it appears to be unclear whether grid cells are the most abundant cell type in the MEC, because almost two times more spatially periodic cells without hexagonal symmetry than grid cells (44 versus 26%) were recorded in a recent study [11]. With all this, it seems that the numeric predictions of our model are well within the range of published findings.

### (h) Anchoring, memory and temporal dynamics

So far, we have not considered here how the grid is anchored to the world. According to our theory, the grid system is a purely metric representation of space, which needs to be aligned with the world, but which does not contain specific spatial memories. The synaptic connections specified in §2*f* may be modified by learning, such that they represent space more accurately (i.e. a kind of procedural memory for distances), but we do not think that grid cells represent environment-specific landmark information. We find it difficult to imagine how individual grid cells could learn landmark information when grid scale is changing with experience, and hence the firing nodes of grid cell activity drift relative to landmark inputs [4]. The type of learning we expect to happen in the grid system is a form of global spatial learning (i.e. a form of learning that is not specific to any one landmark input or any one grid cell), which aligns the entire grid system (and the inherent representation of two-dimensional space) more and more precisely with the specific environment. The decreasing grid scale might be a reflection of such learning [4]. As we already noted, we view this type of learning as a ‘procedural’ learning of distances that occurs incrementally in each environment. Such incremental learning is very different from the instantaneous learning of novel places or contexts in episodic memory formation. Assuming that the grid system has no major role in episodic memory formation might explain why calbindin-positive grid cells do not project to the dentate gyrus of the hippocampus [26] and why the grid system does not show the same extensive remapping in novel environments that is observed in hippocampal place cells [34].

In line with evidence from cognitive psychology [38] and the effect of manipulations of environmental border on hippocampal discharge [39], we expect that the representation of environmental borders plays a critical role in anchoring. Thus, we predict that the critical events in anchoring the entorhinal representation occur in border and band cells [7,8,11] outside of (and downstream from) grid patches. While our model predicts the occurrence of spatial band cells, we do not know whether such band cells can become border cells and what makes border cells stick to certain environmental border cues. Such anchoring of spatial band cells to borders is a critical step in spatial memory formation. We predict that this occurs in stellate cells outside of grid patches. We wonder whether the numerous dendritic spines of stellate cells, which are likely to receive sensory inputs from cortical areas, are the synaptic site and substrate of such learning. Because of their role in anchoring and spatial memory for specific places, it makes sense that stellate cells project to the hippocampus [26]. In order to maintain stable alignment, the results of anchoring also need to be continuously fed back to the grid system, but we do not know how this occurs.

We have also neglected fine temporal dynamics. Such dynamics play a major role in dorsal MEC. We expect that phase precession of grid cells occurs, because the directed propagation of excitation through the grid patch accelerates grid cells relative to theta in the running direction. This issue needs further investigation, however.

## 3. Discussion

We have described a circuit model for grid cell activity. Based on three isomorphism assumptions (isomorphism of neural space in the patch and external space, isomorphism of connection strength and firing field, isomorphism of single cell and population activity), a connectivity scheme for layer 2 grid cells is derived. In this scheme, intrinsic propagation speed in the patch is the neural analogue to spatial scale and is thought to differ roughly 10-fold between dorsal and ventral entorhinal cortex. Head direction input is thought to specify the direction of propagation of activity, but the details of this process are not worked out. We extend the theory to neurons between grid patches and predict the location and discharge properties in these neurons.

### (a) What does the theory explain and predict?

We try to explain the anatomy and the spatial discharge properties in layer 2 of MEC. We did not consider temporal dynamics, the anchoring of the grids to the environment and spatial memory. This account is an explicitly neural/microcircuit hypothesis. The neural nature of the theory implies that it predicts in great detail experimental results. We predict that there is an isomorphism between neural and external two-dimensional space in grid patches. This prediction is in conflict with experimental observations on grid cells in extracellular recordings, which did not show a systematic progression of grid phase in recording tracks [1]. We do not think that this evidence immediately refutes our hypothesis, however, because localization of recorded neurons is imprecise with extracellular recordings and the borders (beginnings/ends) of putative grid patches could not be identified in such recordings. We further predict that grid patches are wired up according to the six rules specified in figure 3 and that activity propagates from cell to cell in two-dimensional space in the patches with a 10-fold higher speed of propagation in dorsal than in ventral cortex. We predict that pyramidal layer 2 neurons in the corresponding positions in neural space—i.e. at the same dorsoventral, mediolateral position of two laterally neighbouring patches—will show temporally correlated activity and aligned grids with the same grid phase in real space. The relative position of grid and ‘band’ cells is also predicted. We predict a relatively rare discharge pattern—inverted grid cells—and their relative spatial position. Ultimate proof for our hypothesis has to come from imaging or electrophysiology experiments.

### (b) Isomorphic computation in cortical networks

The core idea of this paper is that the ‘grid in the world’ (evident in grid cell discharges) is generated by a ‘grid in the head’ (evident in patches in layer 2 of entorhinal cortex). Isomorphic neural representations are evident in the retinotopy of the mammalian visual cortex and the eye-catching representations of body parts such as whiskers [40] or nose appendages [41]. The stunning face representations in somatosensory cortices illustrate the capacity of the mammalian cortex for isomorphic mapping. Such fine-grain ‘body maps’ are often found in tactile specialists suggesting that isomorphic mapping evolved through selective pressure and offers computational advantages. We argue that a ‘grid map’ might benefit from similar isomorphic mapping advantages. Thus, the correspondence of neural and external space will simplify wiring both for grid and body maps. The grid discharge pattern implies anisotropies in spatial representation such as spaces between firing nodes and more firing along spatial directions connecting grid nodes than along spatial directions not connecting grid nodes [3]. Such anisotropies might be more effectively represented in a patchy neural network, where, by definition, connections are also anisotropic across space and directions. Overall, we believe that additional theoretical and experimental work is required to fully grasp the computational advantages of isomorphic mapping.

### (c) Discrete grid topography and patchy architecture

In recordings from the MEC, grid spacing does not appear to increase continuously from dorsal to ventral entorhinal cortex, but it seems to increase in discrete steps [4,5]. We suggest that grid spacing is a shared property of all grid cells in a patch and that discrete steps in grid spacing result from the patchy entorhinal architecture. In simulations of the grid system, it is often difficult to realize multiple grid spacings (R. Kempter 2013, personal communication), and we suggest that the segregated representation of grids in separate subnetworks (patches) might be part of the solution to this problem. This idea is in line with other theoretical studies that also emphasize the role of local interactions in stabilizing the grid structure [42].

### (d) Grid cells outside of layer 2 of medial entorhinal cortex

Grid cells are also found in other layers (e.g. III, V and VI) of MEC [36] as well as other brain areas such as the presubiculum and the parasubiculum [2]. We assume that with respect to the entorhinal cortex the grid is indeed generated in entorhinal layer 2 and simply inherited by the other layers. First, the generation of grid properties in layer 2 is functionally plausible, because a lot of the sensory afferent input to the MEC arrives in layer 1 on the dendrites of layer 2 cells and is not grid-like in nature, whereas many of the layer 2 cells are known to show grid properties. Second, we assume that the other cortical layers simply inherit this property from layer 2. This idea is inspired by findings in cat visual cortex, where the oriented cortical response properties are generated by layer 4 cells from non-oriented thalamic inputs, and it is thought that such orientation selectivity is then inherited by other cortical layers. While this assumption is plausible, we have no strong experimental support for this concept and it is not clear whether input from layer 2 to deeper layers is sufficiently strong to accomplish such a transfer of grid properties.

Third, we see multiple possibilities of how grids could be propagated to the pre- and parasubiculum. It may be the case that these structures also simply inherit grid responses from layer 2 of MEC, but again it is unclear whether the connectivity to accomplish such a transfer of grid properties is present [2]. On the other hand, we find it intriguing that both the presubiculum [43] and the parasubiculum [23] have a strongly modular, patchy structure. Thus, the three structures with prominent grid activity (MEC, pre- and parasubiculum) are all strongly modular in architecture. This may not be co-incidental and we wonder whether all these three structures generate grids through grid patches/modules.

### (e) Comparison with other findings and models

The proposed isoposition connectivity between patches is very similar in phenomenology to the patchy iso-orientation connectivity in visual cortex [44]. The microcircuits of layer 2 in MEC are in some aspects similar in structure to layer 2 microcircuits in retrosplenial cortex [45,46]; it is not clear, however, what functional implications this similarity bears.

Recent intracellular recording [47,48] studies have demonstrated grid-like activity both in layer 2 pyramidal cells and stellate cells. In these studies, cell types have not been ascertained with immuno-histochemical markers such as calbindin and reelin, but nevertheless these findings argue against the strictest versions of our theory, i.e. that the grid system resides exclusively in the calbindin-positive pyramidal cells and that stellate cells are border cells, band cells or inverted grid cells. Recent data from putative stellate neurons identified by optogenetic tagging and back-labelling from the dentate gyrus also argue for the presence of grid cells in the stellate cell population [35]. It is important to understand that, while there are no calbindin-positive pyramidal cells between patches, stellate cells occur both in calbindin patches (where they are a minority of principal cells [26]) and outside of patches (where they are by a wide margin the principal cell type). We therefore think that a slightly modified and generalized version of our hypothesis, in which the grid system resides in grid patches (where it may involve both calbindin and stellate cells) and according to which band and border cells are exclusively stellate cells outside of grid patches, is still compatible with the data.

The fact that we approach grid cell discharge from an anatomical perspective differentiates our hypothesis from previous models [12]. Thus, both interference models [13–17] and attractor models [18–20] typically do not specify microcircuits to the extent that our hypothesis does. The nature of the theory proposed here (with activity propagating through patches that behave like an excitable medium) is clearly closer to attractor models. The hypothesis is aesthetically appealing, because it is very simple and attempts to explain a wide range of phenomena (the whole intricacy of the grid cell system) from very few first principles (the isomorphism assumptions). Given the predictive power of the hypothesis, it can be easily tested.
